# Mechanisms and heterogeneity of in situ mineral processing by the marine nitrogen fixer *Trichodesmium* revealed by single-colony metaproteomics

**DOI:** 10.1038/s43705-021-00034-y

**Published:** 2021-07-13

**Authors:** Noelle A. Held, Kevin M. Sutherland, Eric A. Webb, Matthew R. McIlvin, Natalie R. Cohen, Alexander J. Devaux, David A. Hutchins, John B. Waterbury, Colleen M. Hansel, Mak A. Saito

**Affiliations:** 1 Department of Marine Chemistry and Geochemistry, Woods Hole Oceanographic Institution, Woods Hole, MA USA; 2 Department of Earth, Atmospheric and Planetary Sciences, Massachusetts Institute of Technology, Cambridge, MA USA; 3 Marine and Environmental Biology, Department of Biological Sciences, University of Southern California, Los Angeles, CA USA; 4 Department of Microbiology, Boston University School of Medicine, Boston, MA USA; 5 Department of Biology, Woods Hole Oceanographic Institution, Woods Hole, MA USA; 6 Present Address: Department of Environmental Systems Science, ETH Zürich, Zürich, Switzerland; 7 Present Address: Department of Earth and Planetary Sciences, Harvard University, Cambridge, MA USA

**Keywords:** Biogeochemistry, Microbial ecology, Proteomics, Molecular ecology

## Abstract

The keystone marine nitrogen fixer *Trichodesmium* thrives in high-dust environments. While laboratory investigations have observed that *Trichodesmium* colonies can access the essential nutrient iron from dust particles, less clear are the biochemical strategies underlying particle–colony interactions in nature. Here we demonstrate that *Trichodesmium* colonies engage with mineral particles in the wild with distinct molecular responses. We encountered particle-laden *Trichodesmium* colonies at a sampling location in the Southern Caribbean Sea; microscopy and synchrotron-based imaging then demonstrated heterogeneous associations with iron oxide and iron-silicate minerals. Metaproteomic analysis of individual colonies by a new low-biomass approach revealed responses in biogeochemically relevant proteins including photosynthesis proteins and metalloproteins containing iron, nickel, copper, and zinc. The iron-storage protein ferritin was particularly enriched implying accumulation of mineral-derived iron, and multiple iron acquisition pathways including Fe(II), Fe(III), and Fe-siderophore transporters were engaged. While the particles provided key trace metals such as iron and nickel, there was also evidence that *Trichodesmium* was altering its strategy to confront increased superoxide production and metal exposure. Chemotaxis regulators also responded to mineral presence suggesting involvement in particle entrainment. These molecular responses are fundamental to *Trichodesmium’s* ecological success and global biogeochemical impact, and may contribute to the leaching of particulate trace metals with implications for global iron and carbon cycling.

## Introduction

Marine nitrogen fixation is a key process that stimulates primary production in the N-depleted surface ocean, thereby influencing global carbon and nitrogen cycling [1, 2]. First observed by mariners who referred to it as “sea sawdust,” the colonial cyanobacterium *Trichodesmium* is now recognized to be a major contributor to oceanic nitrogen fixation and therefore a crucial player in global nitrogen and carbon cycling [3, 4]. Due to the high iron requirement of the nitrogenase enzyme, iron (Fe) is hypothesized to be a major control on the distribution of nitrogen fixers, particularly *Trichodesmium* [5]. Many mechanisms for iron uptake and utilization in *Trichodesmium* have been characterized, and the deployment of iron acquisition pathways varies contextually based on chemical and biotic conditions [6–11].

**Fig. 1:**
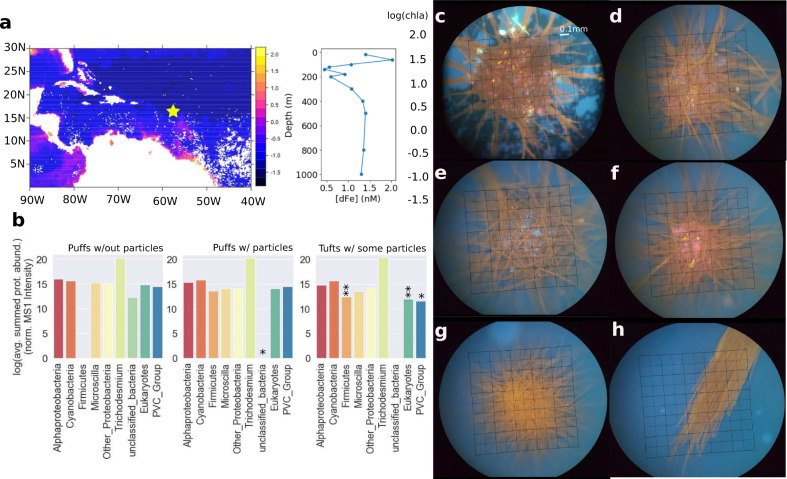
Overview of sampling location and *Trichodesmium* population. **a** Sampling location (yellow star) overlaid on MODIS-Aqua chlorophyll-A data averaged between March 1 and March 31, 2018, with dissolved iron profile at the sampling location. **b** Biological diversity of the proteins identified in the single-colony metaproteomes, with abundances of all detected proteins summed by major taxonomic groupings and separated by colony morphology. * Indicates significant difference from puffs without particles at *p* < 0.1, ** the same at *p* < 0.05, Welch’s unequal variance *t*-test. **c**–**f** Representative images of puff colonies with particles, **g** puff without particles, and **h** a tuft with some particles. Images were collected in epifluorescent mode using a DAPI long-pass filter set, without dyes. The scale bar in (**c**) applies to all images.

In nature, *Trichodesmium* forms large colonies that can reach several millimeters in size, with distinct morphologies including “puffs” and “tufts” (e.g., Fig. 1g, h). These colonies harbor complex microbiomes, also  referred to as epibionts, with diverse heterotrophic and phototrophic bacterial communities [12, 13]. The benefits of colony formation have been debated, but there is renewed interest in their utility for particulate iron acquisition [14–17]. This is important because *Trichodesmium* thrives in regions where iron-rich continental dust is deposited such as the North Atlantic, Red Sea, and near landforms including Australia and the Caribbean islands [18, 19]. Dust addition experiments, both in the laboratory and in shipboard incubations, have demonstrated that *Trichodesmium* puff colonies can selectively capture [20] and efficiently acquire iron from (oxy/hydro)oxide dust minerals [14–16, 21] with the involvement of the epibiont community [22, 23]. Dust particles can relieve iron stress and improve *Trichodesmium’s* metabolic function [24, 25], but may also act as ballast leading to accelerated sinking of *Trichodesmium* colonies and enhanced carbon sequestration [26]. The mechanisms allowing *Trichodesmium* to capture, retain, and dissolve dust particles are therefore of biogeochemical importance.

Here we conducted a combined mineralogical and molecular characterization of individual *Trichodesmium* colonies all collected from a single plankton net conducted at 17:00 local time on March 11, 2018, in the tropical Atlantic Ocean in the vicinity of the Orinoco and Amazon river plumes (−57.5° W 16.5° N, Fig. 1a). Microscopic examination revealed that some, but not all, of the colonies were laden with particulate matter, later confirmed by synchrotron-based element mapping and mineralogical analysis to be iron-oxide and iron-silicate minerals. Subsequent comparative proteomic analysis of individual colonies via a new low-biomass approach revealed key molecular changes including the deployment of proteins involved in iron uptake and storage, redox regulation, heavy metal detoxification, and chemotaxis machinery which may be involved in particle entrainment. These findings indicate there is a distinct in situ molecular response to particulate iron which likely underlies *Trichodesmium’s* competitiveness and biogeochemical function in high-dust surface ocean ecosystems.

## Results and discussion

### Oceanographic context of the sampling location

All *Trichodesmium* colonies used in this study were collected from the same phytoplankton net which sampled a surface-ocean Southern Caribbean Sea community (Fig. 1a). At the sampling station the phosphate concentration was low (0.13 μM at 100 m) as is typical in an oligotrophic environment, while the surface dissolved iron concentration was relatively high (2.02 nM at 100 m), consistent with coastal or atmospheric inputs being mobilized in this region (Fig. 1a). By far the most abundant *Trichodesmium* species at this location was an uncharacterized *Trichodesmium thiebautii* species, as determined by *Trichodesmium*-specific metagenome-assembled-genome recruiting (see Table S1).

Thirty individual colonies of mixed morphology were separated by hand-picking, immediately examined, and photographed by fluorescent microscopy (385 excitation, >420 nm emission), then frozen individually for particle characterization and metaproteomic analysis (Fig. S1). All colonies used in this study presented as healthy with reddish-orange pigmentation and well-defined shape. When the particles were present they auto-fluoresced in the visual light range, appearing as yellow, red, or blue dots. In general, the particles were concentrated in the center of puff-type colonies, though they were also present in tufts but in smaller numbers. Strikingly, colonies either had many such particles or none at all. Based on prior experimental evidence demonstrating that *Trichodesmium* colonies can capture mineral particles and access iron from them [14–16, 21], we hypothesized that these particles were terrestrially derived minerals (Fig. 1c–h). Therefore, we embarked to understand the morphological heterogeneity by characterizing the particles and the colony’s molecular response to them.

### Mineralogical characterization of the colony-associated particles

**Fig. 2:**
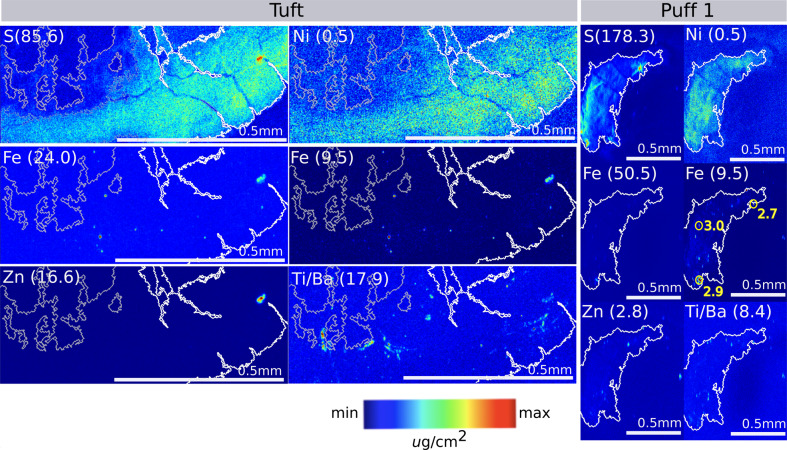
μ-XRF-based element maps of a *Trichodesmium* tuft (left) and puff (right) colony (beamsize 3 ×3 μm). White/gray contours, based on the sulfur panel, which is indicative of biomass, have been provided (white = high [S] threshold, gray = lower [S] threshold). The color scale is the same for each image, with the maximum concentration for each element indicated in parentheses; iron is displayed using two scales. Iron oxidation states were determined via μ XANES for three particles in the puff colony, and these are annotated in yellow. The corresponding XANES spectra are shown in Fig. S4 and tabulated data in Table S2.

To find out whether these natural colonies of *Trichodesmium* had captured iron-rich mineral particles, we performed synchrotron-based micro-X-ray fluorescence (μ-XRF) element mapping of representative colonies with the observed particle associations. Prior evidence of *Trichodesmium*–particle associations has been based mainly on experimental “feeding” of dust to cultured or captured colonies [15, 16, 20, 22–25], and it was therefore important to establish these specific *Trichodesmium*–particle relationships, which developed in nature. We examined one tuft- and two puff-type colonies, all of which had particles associated with them. The element maps were consistent with the hypothesis that there were mineral particles enriched in iron (Fe), copper (Cu), zinc (Zn), titanium/barium (Ti/Ba, which cannot be distinguished by this method), manganese (Mn) and cobalt (Co), though the concentrations approached the limit of detection for the latter two elements (Fig. 2, Figs. S2 and S3). Iron concentrations were particularly high in the particles. Micro-X-ray absorption near-edge structure (μ-XANES) spectra for iron were collected on six particles—three each from the two puffs (Fig. 2 and Fig. S4). The particles contained mineral-bound iron with average oxidation states of 2.6, 2.7, two of oxidation state 2.9, and two of oxidation state 3.0 (Table S2, Fig. S5). While the mineralogy of these particles could not be definitively resolved using μ-XANES, the structure of the absorption edge and post-edge region provided insight into broad mineral groups. Both Fe(III) (oxy/hydro)oxides and mixed-valence iron-bearing minerals consistent with iron silicates were present, suggesting heterogeneous mineral character. While we could not positively identify the silicate mineral phases based on XANES, the spectroscopic similarity of some samples to iron-smectite and the geologic context suggest iron-bearing clays were present (Fig. S5). In this geographic region, iron oxides and clays could be sourced from atmospheric dust deposition, which is common in this region [27, 28] and/or from riverine sources such as the Orinoco and/or Amazon rivers [29, 30].

These colony-associated mineral particles likely serve as a simultaneous source of nutritional (Fe, Ni, Co, Mn) and toxic (Cu) metals to the colonies. The elemental composition of the particles is similar to a recent characterization of *Trichodesmium*-particle associations in the South Atlantic [30]. Release of metals from the particles likely vary over time, with copper, nickel, zinc, and cobalt continually leaching and iron leaching initially, then re-adsorbing back onto particles unless organic chelates assist in solubilization [31].

### Proteome composition is altered by particle presence

To understand the impact of the particles on colony diversity and function, we performed comparative metaproteomic analysis of the individual *Trichodesmium* colonies and their microbiota. Seven puffs without particles, 14 puffs with particles, and 4 tufts with particles were analyzed by a new single-colony metaproteomic method. This approach allowed for the first time the molecular profiles of heterogeneous *Trichodesmium* colonies to be examined individually. Compared to bulk population-level metaproteomes from this location, which achieved deeper resolution of low-abundance proteins by integrating biomass from 50 to 100 colonies (﻿4478 proteins identified) [32], proteome coverage for the low-biomass single colonies was lower yet sufficient for characterizing colony function (2078 proteins identified, Fig. S6) [32]. In total, 1591 *Trichodesmium* and 487 epibiont proteins were identified across the 25 single-colony metaproteomes versus 2944 *Trichodesmium* and 1534 epibiont proteins across triplicate population-level metaproteomes (Tables S3 and S4). Phylogenetic exclusivity was checked such that peptides used to identify epibiont proteins were not present in the *Trichodesmium* genome (Table S5 and Fig. S7) [33, 34].

*Trichodesmium’s* epibiont community plays crucial roles in colony health and physiology, and together the single-colony proteomes demonstrated a diverse and functionally active microbiome associated with the colonies (Fig. 1b and Fig. S8). The proteomic analysis generally reflected the more abundant, “core” members of the epibiont community as was expected given their low-biomass proportion relative to *Trichodesmium* cells. Many commonly identified epibiont groups were present including Alphaproteobacteria, *Microscilla*, and non-*Trichodesmium* cyanobacteria [12, 35, 36]. In general, epibiont abundance was unaffected by particle presence, with one exception: Firmicute proteins were more abundant in tufts and puffs with particles, suggesting enhanced, possibly anaerobic, metabolism. Greater differences were identified between the puff and tuft morphologies, independent of particle presence and consistent with prior characterizations finding that puffs and tufts harbor distinct epibiont communities [12]. Specifically, eukaryotic proteins were more abundant in puffs compared to tufts. These proteins likely represent copepods due to sequence similarity to the model organism *Calanus finmarchicus*, and this result is consistent with observed associations between copepods and puffs at this location (Fig. S8B). Notably, proteins from the PVC superphylum, particularly an uncharacterized eukaryote pathogen species related to *Chlamydia*, were also more abundant in puffs. Eukaryotes are often observed in association with *Trichodesmium* colonies, but are not always identified due to differences in sampling protocols that could wash them away [12], as well as due to biases in analytical methods, for instance in studies with a focus on bacterial 16S or metagenomic analyses. Overall, the differences in the epibiont community were small, suggesting that these do not explain the observed morphological heterogeneity. We therefore turn our attention to describing how the particles impacted the proteome of *Trichodesmium* specifically.

Mineral presence was associated with significant differences in the *Trichodesmium* proteome. In total, 131 proteins were differently abundant in puffs with particles versus without particles (*p* < 0.05, FDR-controlled Welch’s unequal variances *t*-test). Proteome differences were distributed across a variety of biogeochemically relevant proteins, particularly metalloproteins containing iron, nickel, copper, and zinc. Photosynthesis and carbon fixation proteins including Rubisco (*p* = 0.04), citrate synthase (*p* = 0.08), and the accessory pigment allophycocyanin (*p* = 0.1) were more abundant when minerals were present. In fact, most of the differentially abundant proteins were more abundant in the particle-containing colonies suggesting that the colonies with particles were more metabolically active. Particle presence did not affect nitrogenase abundance, consistent with prior laboratory experiments in which *Trichodesmium erythraeum* filaments were fed concentrated dust [24]. Because nitrogen fixation is a critical process, *Trichodesmium* may distribute iron to nitrogenase at a steady rate, while altering the activity of other systems in response to the particles. Alternatively, it is possible that nitrogenase activity was being regulated post-translationally [37] and there were indications of enhanced use and recycling of fixed nitrogen compounds; the nitrogen assimilation proteins glutamine synthetase (*p* = 0.008), spermidine synthase (*p* = 0.005), and a urea transporter (*p* = 0.02) were enriched in colonies with particles (see Fig. S9) [32].

### Responses in iron uptake and utilization proteins

**Fig. 3:**
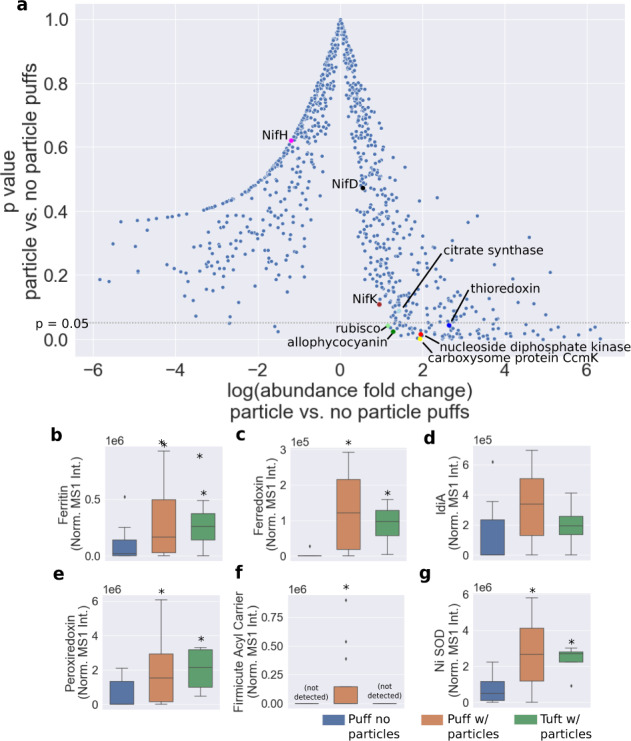
Proteome responses in colonies with and without particle associations. **a** *p* value (Welch’s *t*-test) versus protein abundance reported as fold change for puffs with vs. without particles. Only *Trichodesmium* proteins are displayed. Below the gray dotted line (*p* = 0.05), the differences are statistically significant. Positive fold change indicates the protein was more abundant when particles were present. Proteins of interest are highlighted. **b**–**g** Relative abundance of selected proteins for the different colony types, presented as box plots (center line = median, box limits = first and third quartiles, whiskers = data min and max, diamonds = outliers). *Indicates statistically significant difference compared to the puffs without particles, *p* < 0.05.

The broadest and strongest proteome response occurred in iron-related proteins, consistent with *Trichodesmium’s* strong dependence on iron availability. Several iron-containing proteins were significantly more abundant when particles were present, suggesting that the particles acted as a micronutrient source. These included an iron-containing peroxidase (*p* = 0.03), the electron transport protein ferredoxin fdxH (*p* = 0.0006), and the iron storage/DNA binding protein Dps-ferritin (*p* = 0.002) (Fig. 3). Together, the coordinated response of each of these proteins suggests that the minerals were a nutritional source of iron. For instance, increased abundance of ferredoxin is consistent with increased iron availability, since the non-iron-containing flavodoxin is substituted for ferredoxin during iron stress [6, 38, 39]. Similarly, increased abundance of Dps-ferritin would serve to buffer and store iron acquired from the concentrated particulate metal source. In addition to binding iron, the Dps-ferritin may also serve to protect DNA from photooxidative damage, and this in addition to the peroxidase signal may indicate increased oxidative stress in the colonies with particles [40]. The clear increase in Dps-ferritin protein abundance in the presence of mineral particles differs from a prior report, which found no change in bacterioferritin transcript abundance when cultured *T. erythraeum* was fed Saharan desert dust [24]. It is possible that Dps-ferritin and bacterioferritin respond to distinct cellular conditions, for instance Dps-ferritin may have been preferred by these colonies because of its dual function for iron storage and protection against oxidative stress. This difference highlights the potential challenges in comparing laboratory and field studies, as well as the often-suggested importance of post-transcriptional and post-translational controls in *Trichodesmium* [32, 37, 41]. Given the high iron demand of the nitrogenase metalloenzyme, the ability to store iron from rich but episodically available mineral particles could provide an important ecological niche in oceanic environments where iron can be scarce and its solubility is low.

**Fig. 4:**
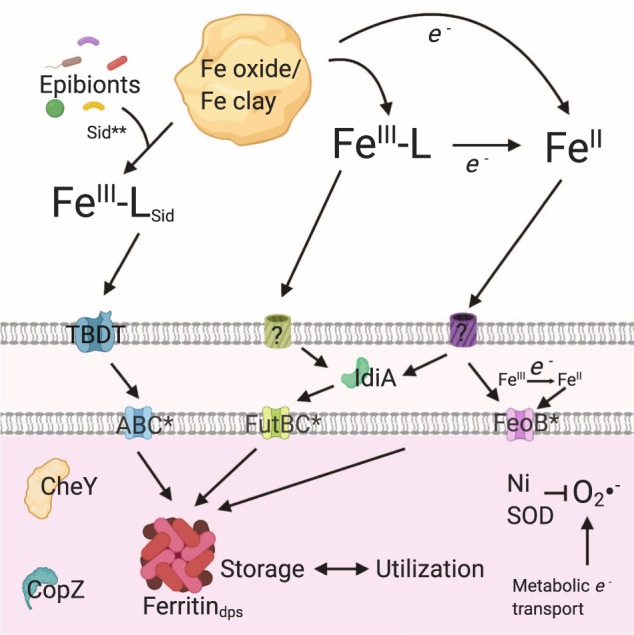
Current model of iron uptake and use mechanisms involved in utilization of particle-derived iron, based on mineralogical and proteomic observations in this and prior studies. Fe(III)-L and Fe(II) are thought to enter the periplasm through unknown passive porins/receptors (green and purple proteins labeled “?”) [9]. Iron acquisition proteins with annotated names are identified, otherwise the following general functional names are used: TBDT unspecified TonB dependent transporter, ABC unspecified ABC transporter, Ni SOD nickel superoxide dismutase, CheY chemotaxis response regulator, CopZ copper chaperone. IdiA preferentially binds iron in the Fe(III) state but may also bind Fe(II) [11]. *e*- indicates any general reductant, which could include extracellular superoxide. *Indicates iron acquisition proteins that were not identified in this study. **Evidence from this study suggests that the Firmicute epibionts were producing siderophores.

Multiple uptake mechanisms were involved in obtaining iron from the mineral particles. While iron acquisition in *Trichodesmium* is not well characterized, at least three systems are known to exist in the genome: the FeoB system (Fe(II)), the IdiA system (Fe(III)), and uptake via Fe-siderophores [9, 11]. The single-colony metaproteomes provided evidence for the deployment of the latter two mechanisms in the natural environment; FeoB is rarely identified in field metaproteomes of diazotrophs, possibly due to its low copy number and high efficiency (Fig. 4) [38, 39, 42]. Despite evidence that the particles provided iron to the colonies, the periplasmic iron transport protein IdiA was more abundant during particle associations (*p* = 0.16) (Fig. 3d). IdiA is often used as a biomarker of iron stress because it is responsive to/more abundant in low iron conditions [32, 38, 39]. Increased IdiA abundance was confusing because it suggested that the particle-associated colonies were more iron limited, a conclusion that is not supported by the increase in iron storage and utilization proteins described above. One interpretation is that IdiA serves a function in acquiring iron from the mineral particles. Due to its binding preference for Fe(III) [11] this could include uptake from a ligand-bound Fe(III) state that may aid in particle dissolution [11, 43, 44]. In this way, IdiA may have a different functional response to particulate versus dissolved iron. There was also evidence that iron-binding siderophore systems synthesized by the bacterial epibionts were involved: a Firmicute acyl carrier protein putatively involved in siderophore production was enriched in puffs with particles (Fig. 3f, *p* = 0.04). *Trichodesmium* does not seem to produce siderophores, but it can acquire siderophore-bound iron produced via mutualistic interactions with epibionts, especially when provided with concentrated dust [9, 22, 23, 43]. Corroborating this, a *Trichodesmium* TonB-dependent transporter (TBDT) for ferrienterochelin/colicins was identified only in puffs with particles [45].

### A crucial role for redox regulation when particles were present

The enzyme nickel superoxide dismutase (Ni SOD) was significantly more abundant in the mineral-associated colonies (Fig. 3g*, p* = 0.004), reflecting the need to regulate the reactive oxygen species (ROS) superoxide in the presence of particles. There are multiple reasons for elevated superoxide production in particle-associated colonies. First, metabolic electron transport is associated with incidental superoxide production, and electron transport seems to have been enhanced in the presence of particles as indicated by the enrichment of photosynthesis proteins. Furthermore, *Trichodesmium* has among the highest Mehler reaction activity of any photosynthetic organism, and this may contribute to internal superoxide production [46]. Indeed, this evidence of an abundant ROS detoxification enzyme is consistent with intracellular superoxide production and indicates that the traditional Mehler reaction is important in field populations. Additional redox-regulating proteins including peroxiredoxin, thioredoxin, and Dps-ferritin were more abundant when particles were present (see Fig. 3e, *p* = 0.03 and 0.04, respectively, for puffs with and without particles), consistent with elevated ROS in particle-containing colonies. Increased superoxide dismutase protein may also reflect enhanced nickel availability when particles were present. Nickel is an essential nutrient for *Trichodesmium*, and can limit nitrogen fixation due to *Trichodesmium’s* significant need for superoxide dismutase to protect the nitrogenase enzyme [47].

An additional explanation for increased superoxide dismutase protein abundance is that it indirectly reflected iron uptake processes and/or extracellular iron reduction. Reduced iron transported into the cell could react with intracellular oxygen, leading to increased superoxide formation; [48] this could trigger superoxide dismutase production to maintain healthy intracellular superoxide levels [49]. Puffs in particular are known to closely regulate superoxide levels with a possible link to cell signaling and growth [50]. Extracellular superoxide production may also play a role in iron uptake, as has been suggested previously [9]. This extracellular superoxide production may indirectly contribute to intracellular superoxide dismutase signals via Fe(II) uptake as described above, but because superoxide does not cross cell membranes, this does not directly explain the observed signal in intracellular superoxide dismutase [51]. Besides extracellular superoxide, other mechanisms of reductive iron uptake have been suggested including reactive metabolites and extracellular proteins on the cell surface [9]. Recent observations have tied hydrogen physiology to mineral iron uptake, however, the underlying biochemical system remains unknown [21]. Systems such as the alternative respiratory terminal oxidase (ARTO) have been invoked [52], but this protein was not detected, implying it was  not a major constituent of the proteome, though as in the above case of FeoB it is possible that ARTO was present in low concentrations and  was highly active. The putative role of ARTO is furthermore unclear because a recent study observed that ARTO transcript abundance was lower in the presence of dust, and not enhanced as might be expected if ARTO is involved in iron uptake [24].

### Involvement of chemotaxis machinery in particle entrainment

A prior study captured impressive visual observations of *Trichodesmium* responses to dust, where experimentally provided particles were captured and transported to the colony center [15]. This behavior was seen primarily in puff-type colonies isolated from the field, indicating that these natural populations may react differently to dust than laboratory *Trichodesmium* strains. Consistent with prior studies [15, 20], the mineral particles were concentrated in the center of puff-type colonies and this may enhance the efficiency of extracellular iron reduction pathways by reducing diffusive loss [53]. Moreover, the proteomic characterization from this study provides molecular insight into this behavior. Here we observed that movement proteins including a SwmA-like RTX protein (*p* = 0.1) [54] and chemotaxis regulator CheY (*p* = 0.3) were enriched when particles were present (Fig. S9E–H), indicating that they were involved in the particle capturing behavior observed by Rubin et al. [15]. In previously published bulk metaproteomes of field *Trichodesmium* populations, there was an observed relationship between the CheY protein and the iron transport protein IdiA, suggesting a link between chemotaxis and particulate iron acquisition [32]. While there is more to be learned about how this system operates, it appears that the chemotaxis and iron regulatory systems are connected and responding directly to particulate iron.

### Evidence for a specific regulatory response to particulate iron in *Trichodesmium*

Taken together, the in situ biochemical observation points to a model in which *Trichodesmium* colonies differentiate between dissolved versus particulate iron. First, consistent with the mineralogical profile of the particles, the colonies engaged multiple iron uptake mechanisms and also prioritized balancing cellular redox status during enhanced productivity and iron uptake (Fig. 4). Once inside the cell, mineral-derived iron was preferentially stored via Dps-ferritin. This finding adds complexity to the canonical regulatory model that IdiA and ferritin exist on a continuum with IdiA abundant during iron limitation and ferritin present only when iron is replete. It suggests that *Trichodesmium* alters uptake and utilization mechanisms in response to the iron’s coordination environment, specifically to the particulate vs. dissolved phase. Whether *Trichodesmium* makes the distinction directly via a specific mineral sensing mechanism, or indirectly due to increased intracellular metal concentration, is not yet known. *Trichodesmium* has an unusually large number of uncharacterized two-component sensory systems relative to other phytoplankton, and it is possible that one or more of these systems is involved, which would further imply the involvement of metal-binding ligands [55]. Either way, it is clear that multiple metabolic and homeostasis systems were involved in this coordinated response to the presence of particulate iron, and it does seem likely that iron-specific regulatory processes are involved since in laboratory studies *Trichodesmium* is able to distinguish between iron-containing and non-iron-containing particulate matter [20]. A final intriguing aspect of particle-associated *Trichodesmium* colonies was the deployment of Cu-related proteins. *Trichodesmium* is known to be extraordinarily sensitive to Cu toxicity [56], and close proximity to mineral particles was associated with enrichment of the copper chaperone/homeostasis protein CopZ (*p* = 0.06 and Fig. S9D) [57], suggesting the need for metal detoxification when minerals were present.

### Conclusion

This study provides direct evidence that *Trichodesmium* colonies capture and process oxide and silicate minerals in situ, and that colonies alter key aspects of their biochemistry in response. These findings highlight heterogeneity in the morphological and molecular profiles of *Trichodesmium* colonies in nature, revealed by a new approach in which colony proteomes are analyzed individually. The underlying mechanisms for this heterogeneity are multiple and likely intersect. One important aspect is stochastics of colony-particle encounters due to patchy dust availability. In addition, the sampled population might represent a mixture of *Trichodesmium* colonies advected from geographical locations or water column environments/depths with different particle loadings. Other explanations could include sub-species differences among the colonies, unresolved changes in the epibiont community, and past physiological history of the colony such as age or past iron status. Clearly much is still to be discovered about the reasons for and implications of physiological heterogeneity in natural marine microbial populations.

*Trichodesmium* thrives in high-dust environments and this study provides clear evidence for the biochemical basis behind this specialized niche. The results have ecological and geochemical implications beyond *Trichodesmium* biology. Specifically, active capture and degradation of mineral particles may increase iron availability in the oligotrophic surface ocean. In this way, abundant *Trichodesmium* colonies may have an important role in the leaching of particulate trace metals and the supply of bioavailable iron to the euphotic zone, not least because *Trichodesmium* colonies tend to be neutrally or positively buoyant [58, 59] and can therefore retain mineral matter at the surface. Mineral capture and degradation may therefore have significant implications for global iron and carbon cycling [60]. Furthermore, the coupled mineralogical and biochemical characterization of single colonies present evidence for specific responses to mineral particles that could be leveraged to improve future biogeochemical and marine ecosystem models. Particulate iron utilization by *Trichodesmium* appears to be a critical niche, and is likely a significant factor determining this organism’s ecological success and fixed nitrogen contributions to the global ocean.

## Materials and methods

### Sampling and microscopy

All of the colonies used in this study were sampled from a single plankton net conducted at −57.5° W 16.5° N at 17:00 local time on March 11, 2018, on the AT39-05/Tricolim expedition (R.V. Atlantis, Chief Scientist D. Hutchins, https://www.bco-dmo.org/deployment/765978). A 130-μm net was released to approximately 20 m depth, then pulled back to the surface and the process repeated five times. Colonies were handpicked by gentle pipetting, rinsed twice in 0.2-μm-filtered trace metal clean seawater, and decanted into 0.2-μm sterile filtered trace metal seawater until imaging. All at-sea colony picking and handling was conducted in a Class 100 trace metal clean environment. Colonies were imaged with a Zeiss epifluorescent microscope using transmitted light and/or a long-pass fluorescent filter set. At the time of imaging, they were labeled as “particle containing” or not and classified as puffs or tufts. They were then decanted individually onto trace metal clean 0.2-μm Supor filters and flash frozen in liquid nitrogen, then stored at −80 °C until analysis at the home laboratory. Images were captured with a Samsung Galaxy Note 4 using a SnapZoom universal digiscoping adapter. MODIS-Aqua data for Fig. 1a was obtained from the NASA Goddard Space Flight Center, Ocean Ecology Laboratory, Ocean Biology Processing Group; (2014): Sea-viewing Wide Field-of-view Sensor (SeaWiFS) Ocean Color Data, NASA OB.DAAC Accessed on 2020-04-08.

### Nutrient and trace metal analyses

Seawater was sampled using a trace metal clean rosette consisting of Niskin-X bottles. Niskins were pressurized with nitrogen gas in a shipboard Class 100 clean room and seawater was filtered through 0.2-μm Supor membranes to remove particles. Aliquots for macronutrient (phosphate) analysis were frozen immediately at sea and were thawed just prior to analysis. Phosphate was quantified using a Technicon Autoanalyzer II by Joe Jennings at Oregon State University. Aliquots for dissolved metal analysis were acidified with concentrated trace metal clean HCl (Seastar) to pH 1.8 and allowed to equilibrate for ~1 month prior to analysis. Dissolved iron was concentrated using a seaFAST automated pre-concentration system and quantified on an ICAP Q inductively coupled plasma mass spectrometer (ICP-MS).

### Sample handling and preparation for single-colony proteomics

Upon return to the lab, the colonies were carefully cut out of the filter to reduce the volume of liquid needed for protein extraction. The filter sections were submerged in PBS buffer with 1% sodium dodecyl sulfate (SDS), 1 mM magnesium chloride, 2 M urea, heated at 95 °C for 10 min, then shaken at room temperature for 1 h. The extracts were then treated with benzonase nuclease for 30 min at 37 °C. Proteins in the resulting supernatant were quantified by the BCA assay. Total protein content varied from <1–10 μg among the colonies, and scaled with colony size. The proteins were digested with a modified polyacrylamide tube gel protocol following Saito et al. 2014 in part to purify the protein biomass from  any particulate matter Instead of the typical 200 μL final gel volume only 50 μL final volume was used to minimize protein loss [61, 62]. In addition, the protein precipitation/purification step was eliminated because this is another source of total protein loss. Instead, the samples were treated with benzonase nuclease during the initial extraction phase to solubilize any DNA/RNA components, allowing the purification step to be skipped. Briefly, the proteins were embedded in an acrylamide gel, washed with a 50:50 acetonitrile: 25 mM ammonium bicarbonate buffer, dehydrated by acetonitrile treatment, then treated for 1 h at 56 °C with 10 mM dithiothreitol in 25 mM ammonium bicarbonate followed by 1 h at room temperature with 55 mM iodacetamide. Gels were dehydrated again and rehydrated in trypsin (Promega Gold) at a ratio of 1 μg trypsin: 20 μg total protein in 25 mM ammonium bicarbonate. Proteins were digested overnight at 37 °C with shaking. The peptides were then extracted from the gels in 20 μL peptide extraction buffer (50% acetonitrile, 5% formic acid in water). The resulting peptide mixtures were concentrated to 0.2 μg total protein/μL final concentration. While the tube gel method was used for samples presented here, magnetic bead and soluble protein digestion methods were also tested. Total protein recovery was lower with these methodologies, perhaps because these methods do not use SDS, which in our hands is a good lysing agent for *Trichodesmium*.

### LC–MS/MS analysis

Metaproteome analyses were conducted by tandem mass spectrometry on a Thermo Orbitrap Fusion using 0.5 μg total protein injections and a one-dimensional 120 min non-linear gradient on a 15 cm C18 column (100 µm × 150 mm, 3 µm particle size, 120 Å pore size, C18 Reprosil-Gold, Dr. Maisch GmbH packed in a New Objective PicoFrit column). LC lines were shortened when possible to reduce the possibility of sample loss to the tubes. Blanks were run between each sample to avoid carryover effects. For each run 0.5 μg of protein was injected directly the column using a Thermo Dionex Ultimate3000 RSLCnano system (Waltham, MA); if less than 0.5 μg of protein was available, the entire sample was injected. The samples were analyzed on a Thermo Orbitrap Fusion mass spectrometer with a Thermo Flex ion source (Waltham, MA). MS1 scans were monitored between 380 and 1580 *m*/*z*, with a 1.6 *m*/*z* MS2 isolation window (CID mode), 50 ms maximum injection time and 5 s dynamic exclusion time. The resulting spectra have been deposited to the ProteomeXchange Consortium via the PRIDE partner repository with the dataset identifier PXD016330 and 10.6019/PXD016330 [63].

### Bioinformatics analyses

The spectra were searched using the SEQUEST algorithm with a custom-designed *Trichodesmium* sequence database composed from a publicly available *Trichodesmium* metagenome, trimmed based on a prior bulk metaproteomic analysis, and location-specific metagenome-assembled genomes (MAGs) of *Trichodesmium* and the epibiont community (Fig. S10). To trim the metagenome sequence database, triplicate Tricho-enriched bulk metaproteomes from the same location (aka “population biomass” samples), each integrating ~50 to 100 colonies handpicked from the same plankton net, were analyzed using a publicly available *Trichodesmium* consortia metagenome collected at Station BATS (IMG ID 2156126005). All proteins identified at the 1% protein and peptide FDR level (Scaffold software, Proteome Software, Inc) were then carried forward into the custom-designed sequence database. Next, to improve coverage of the specific sampled population, hand-refined metagenome-assembled genomes from *Trichodesmium* populations throughout the AT39-05 transect were also added to the database: these included four *T. thiebautii* species (one H94 species and three uncharacterized *T. thiebautiis*) and 17 MAGs from the epibiont community.

The single-colony metaproteomes were searched using the SEQUEST search engine with parent mass tolerance ±10 ppm and fragment mass tolerance 0.8 Dalton, allowing cysteine modification of +57.022 Daltons and methionine modification of +16 Daltons. The results were statistically validated at the 1% FDR level using the Scaffold program (Proteome Software, Inc).

The custom-designed sequence database was crucial in improving coverage of the proteome at the single-colony level. When the data were searched with the entire *Trichodesmium* consortial metagenome (IMG ID 2156126005, 240447 protein-encoding genes), only 800 proteins were identified at the 1% protein and peptide FDR level due to mis-match of the sequence database size to the proteome diversity of the sample [64, 65]. Trimming the search space to focus on proteins identified in the bulk metaproteome analysis (reducing the sequence database to 4478 proteins) nearly doubled the number of proteins identified, to 1495 proteins. Including the location-specific MAGs enhanced the coverage further, with 2078 proteins being finally identified in the final search using the final custom database.

Peptides used to identify *Trichodesmium* proteins were determined to be phylogenetically exclusive to the genus using the open source Metatryp software package last accessed on May 23, 2020 [33, 34]. Statistical tests (Welch’s t tests) were performed using the Scipy stats python library and the results are reported in Table S1. *P* values were FDR controlled by the Benjamini–Hochberg procedure; at *α* = 0.05 and *α* = 0.1, the calculated FDR was 0%; the FDR rose to 0.56% by *α* = 0.25.

### Micro-X-ray fluorescence and Micro-X-ray absorption spectroscopy

μ-XRF and micro-X-ray absorption spectroscopy (μ-XAS) were conducted at the Stanford Synchrotron Radiation Lightsource on beamline 2–3 with a 3 μm raster and a 50 ms dwell time on each pixel. μ-XRF data were analyzed using MicroAnalysis Toolkit [66]. Elemental concentrations were determined using standard foils containing each element of interest. The relative proportions of Fe(II) and Fe(III) were determined by fitting the edge position of the background subtracted, normalized XANES spectra. Fe XANES spectra were fit using the SIXPACK Software package [67], and redox state was estimated by conducting a linear combination fitting of the absorption edge (7115–7140 eV) using the model compounds siderite (FeCO_3_) and two-line ferrihydrite as end-member representatives of Fe(II) and Fe(III), respectively. Further, these values were confirmed through deconvolution of the edge shape using Gaussian peaks at two fixed energies corresponding to primary Fe(II) (7122 eV) and Fe(III) (7126 eV) contributions (PeakFit software, SeaSolve Inc.) [68]. Linear combinations of the empirical model spectra were optimized where the only adjustable parameters were the fractions of each model compound contributing to the fit. The goodness of fit was established by minimization of the R-factor [69, 70].

Although mineral identity cannot be conclusively determined with XANES, visual comparison of the edge features are indicative of broad Fe-bearing mineral groups including many common oxide and silicate minerals. Thus, to get a general sense of mineral groups, we have included several of the top spectral library query hits (Fig. S4) for the six particles we looked at in this study. These include two-line ferrihydrite and goethite (as Fe oxyhydroxide phases), ferrosmectite (as Fe-bearing secondary clays), and biotite (as a primary silicate). We also included siderite (as an Fe(II)-bearing carbonate) in Fig. S4 for comparison to a pure Fe(II) phase.

### Image contour analysis for element concentration maps

Image contours were generated for the sulfur element concentration maps in Fig. 2 using the Scikit-image python library [71]. First, using the contrast histograms for the sulfur images, algorithmically defined thresholds were applied. For the tuft image in Fig. 2, two thresholds were used to capture the high and low-biomass regions. The resulting binary images were then morphologically dilated to remove noise and connect gaps between objects. A list of contours for the binary image was generated using the marching squares/cubes algorithm [72]. The longest contours were overlaid on the element maps from which they were generated.

## Supplementary information

Supplementary Material

Supplementary Table S1

Supplementary Table S3

Supplementary Table S4

Supplementary Table S5

## Data Availability

All data are provided in the main text or as supplementary materials. In addition, the mass spectrometry proteomics data have been deposited to the ProteomeXchange Consortium via the PRIDE partner repository with the dataset identifier PXD016330 and 10.6019/PXD016330. The processed proteomics data can also be accessed at BCO-DMO (10.26008/1912/bco-dmo.786694.1).

## Code availability

Data analyses and plotting were conducted in Python 3.0 (https://www.python.org/) using the pandas (https://pandas.pydata.org/), matplotlib (https://matplotlib.org/), seaborn (https://seaborn.pydata.org/), scipy stats (https://scipy.org/), and scikit-image (https://scikit-image.org/) libraries. Fully reproducible example code, including statistical analyses, can be found at https://github.com/naheld/Tricho_Singlecolony_MetaP.
